# Assigning Priorities for Fixed Priority Preemption Threshold Scheduling

**DOI:** 10.1155/2015/837472

**Published:** 2015-11-25

**Authors:** Saehwa Kim

**Affiliations:** Department of Information Communications Engineering, Hankuk University of Foreign Studies, Yongin-si, Gyeonggi-do 449-791, Republic of Korea

## Abstract

Preemption threshold scheduling (PTS) enhances real-time schedulability by controlling preemptiveness of tasks. This benefit of PTS highly depends on a proper algorithm that assigns each task feasible scheduling attributes, which are priority and preemption threshold. Due to the existence of an efficient optimal preemption threshold assignment algorithm that works with fully assigned priority orderings, we need an optimal priority assignment algorithm for PTS. This paper analyzes the inefficiency or nonoptimality of the previously proposed optimal priority assignment algorithms for PTS. We develop theorems for exhaustively but safely pruning infeasible priority orderings while assigning priorities to tasks for PTS. Based on the developed theorems, we correct the previously proposed optimal priority assignment algorithm for PTS. We also propose a performance improved optimal priority assignment algorithm for PTS proving its optimality. The empirical evaluation results clearly show the effectiveness of the proposed algorithm.

## 1. Introduction

Preemption threshold scheduling (PTS) is an extension of preemptive fixed priority scheduling where each task has an extra scheduling attribute, called a preemption threshold, in addition to a priority. The preemption threshold of a task is its run-time priority, which is maintained after the task is dispatched and until it terminates its execution, so it regulates the degree of “preemptiveness” in fixed priority scheduling [1]. If the threshold of each task is the same as its original priority, then PTS is equivalent to fully preemptive fixed priority scheduling (FPS), and if each task has the highest threshold value in a system, it is equivalent to nonpreemptive scheduling (NPS). The use of PTS is very effective in system tuning processes since it enhances real-time schedulability, eliminates unnecessary preemptions, reduces memory stack usage [2] via the notion of nonpreemption groups [3], and allows for scalable real-time system design [4–6]. Preemption thresholds and nonpreemption groups are also parts of OSEK [7] and AUTOSAR [8] standards of automotive operating systems. As remarked in [9], PTS represents an example of a great success of transferring academic research results to industrial applications [10–12].

The benefit of enhanced real-time schedulability of PTS highly depends on a proper algorithm that assigns each task feasible scheduling attributes, which are priority and preemption threshold. The work of this paper has been highly motivated by our previous work of SISAtime [13], which adopts PTS to schedule active (concurrent) objects of real-time object-oriented models [14–19]. While SISAtime contains an optimal scheduling attributes assignment algorithm for PTS, it is not so much efficient. A scheduling attributes assignment algorithm is* optimal* if it is guaranteed to output a feasible (schedulable) scheduling attributes assignment if one exists [1, 20–22].

There are two previously proposed optimal scheduling attributes assignment algorithms for PTS: TRAVERSE( ) of SISAtime [13] and SEARCH( ) of [1], which is the first academic article that presented PTS. Both algorithms work in two stages. At the first stage, priorities are assigned to all tasks. At the second stage, preemption thresholds are assigned using the optimal preemption threshold assignment algorithm, OPT-ASSIGN-THRESHOLD( ) of [1], which has the complexity of *O*(*n* ^2^). Recently, [23] extends OPT-ASSIGN-THRESHOLD( ) by considering the cache-related preemption delay (CRPD), and it also assigns optimal preemption thresholds for tasks with preassigned priorities. Since preemption thresholds are wholly assigned at the second stage, both algorithms focus on how to assign priorities to tasks. With this, we call these optimal “scheduling attributes” assignment algorithms for PTS as optimal “priority” assignment algorithms for PTS.

In this paper, we analytically show that TRAVERSE( ) is inefficient and SEARCH( ) is not optimal. We develop theorems for exhaustively pruning infeasible priority orderings without harming the optimality of priority assignment algorithms for PTS. Specifically, we develop following lemmas and theorems under PTS:

- Under PTS, if the priority of a task is fixed, its worst-case response time does not decrease when its preemption threshold is lowered (Lemma 3).
- Under PTS, if the preemption threshold of a task is fixed, its worst-case response time does not decrease when its priority is lowered (Theorem 6).
- Under PTS, if a task with the highest preemption threshold in a priority ordering is infeasible, the task set with the priority ordering is also infeasible (Theorem 4).
- Under PTS, if a task with the highest preemption threshold in a priority ordering is infeasible, the task set with another priority ordering that assigns the task the lowered priority is also infeasible (Theorem 7).

By applying these theorems, we correct SEARCH( ) and propose CORRECTED-SEARCH( ) which is more efficient than TRAVERSE( ). We also propose PRUNED-TRAVERSE( ) that improves the performance of CORRECTED-SEARCH( ) and proves its optimality.

We also empirically evaluate the performances of the discussed optimal priority assignment algorithms. We first empirically show the usefulness of the proposed optimal priority assignment algorithm by showing that they always achieve the better schedulability than any other existing nonoptimal priority assignment algorithms. We also compare the actual runtimes for executing each optimal priority assignment algorithm as well as PA-DMMPT( ) by [24], which is the most effective heuristic priority assignment algorithm for PTS if it is combined with the policy of deadline monotonic priority ordering (DMPO). The empirical results clearly show that the actual runtimes of TRAVERSE( ) are reduced by CORRECTED-SEARCH( ), whose actual runtimes are also more reduced by PRUNED-TRAVERSE( ) while such performance improvements become drastically large as the number of tasks increases. It is also shown that the actual runtimes of PRUNED-TRAVERSE( ) are even smaller than those of PA-DMMPT( ).

The remainder of the paper is composed as follows.  Section 2 gives the task model with notations and presents a walk-through example that motivates our work.  Section 3 analyzes previously proposed optimal priority assignment algorithms for PTS.  Section 4 corrects previously proposed SEARCH( ) algorithm making it an optimal priority assignment algorithm for PTS.  Section 5 describes our proposed optimal priority assignment algorithm for PTS and proves its optimality.  Section 6 considers the complexity of the discussed optimal priority assignment algorithms.  Section 7 shows our empirical evaluation results. Finally, Section 8 concludes the paper.

## 2. Task Model

We use the same task model as the one used in the traditional preemption threshold scheduling [1, 3, 25, 26]. Specifically, a system has a fixed set of tasks Γ = {*τ*_1_, *τ*_2_,…, *τ*_|Γ|_}. Each task *τ*_*i*_ has a fixed period *T*_*i*_, a fixed relative deadline *D*_*i*_, and a known worst-case execution time *C*_*i*_. There is no restriction such that each task's deadline should be shorter than its period. We also adopt the “integer time model” of [9], where all timing parameters are assumed to be nonnegative integer values.

Each task *τ*_*i*_ also has a fixed priority *p*_*i*_ and a preemption threshold pt_*i*_ where *p*_*i*_ is assigned by a specific priority assignment algorithm and pt_*i*_ is assigned by OPT-ASSIGN-THRESHOLD( ) of [1]. Each task has a distinct priority value: every task has a different priority value. Each task set Γ has |Γ|! distinct priority orderings for its tasks. Accordingly, the set of distinct priority orderings has cardinality |Γ|!, which we denote PO^Γ^ = {PO_1_, PO_2_,…, PO_|Γ|!_}. We denote the resultant priority ordering generated by a specific priority assignment algorithm ALGORITHM( ) as PO_*A*_. With this, a specific priority ordering PO_*n*_ is a sequence of priorities for tasks in Γ, which we denote as PO_*n*_ = 〈*p*_1_^*n*^, *p*_2_^*n*^,…, *p*_|Γ|_^*n*^〉.  The inverse mapping of each priority ordering PO_*n*_ is a task ordering from the lowest priority to the highest priority, which we denote as PO_*n*_^−1^ = TO_*n*_ = 〈*i*, *j*,…, *k*〉 where each number represents a task index. We also denote the inverse mapping of task ordering TO_*n*_ as TO_*n*_^−1^ = PO_*n*_.

We denote a higher priority with a larger value: 1 is the lowest priority value and |Γ| is the highest priority value. Note that it is meaningful to assign a task a preemption threshold that is no less than its regular priority since a preemption threshold is used as an effective run-time priority to control unnecessary preemptions [1]: which means that ∀*τ*_*i*_, pt_*i*_ ≥ *p*_*i*_. Notation section summarizes the notations and associated descriptions used in this paper.

### 2.1. Feasibility Analysis

As the feasibility test under PTS, we adopt the worst-case response time analysis equations of [9]. The original equations were introduced by [1] and their errors were fixed by [27]. These results were refined by [26], whose results in turn were concisely arranged by [9]. We rewrite the relevant equations of [9] for calculating the worst-case response time *R*_*i*_ of task *τ*_*i*_ as follows:

(1) Ri=maxq∈1,Qi⁡Fi,q−q−1·Ti,

(2) Qi=LiTi,

(3) Li=Bi+∑∀j,pj≥piLiTj·Cj,

(4) Fi,q=Si,q+Ci+∑∀j,pj>ptiFi,qTj−1+Si,qTj·Cj,

(5) Si,q=Bi+q−1·Ci+∑∀j,pj≥pi1+Si,qTj·Cj,

(6) Bi=max⁡Cj−1 ∣ ∀j,  ptj≥pi>pj,

where *L*_*i*_ is the longest level-*p*_*i*_ busy period [28], *q* is the index of instances of task *τ*_*i*_ within *L*_*i*_, *Q*_*i*_ is the last index of instances of task *τ*_*i*_ within *L*_*i*_, *F*_*i*,*q*_ is the finish time of the *q*th instance of task *τ*_*i*_, *S*_*i*,*q*_ is the start time of the *q*th instance of task *τ*_*i*_, and *B*_*i*_ is the worst-case blocking time of task *τ*_*i*_. Whenever a variable appears on both sides of an equation (i.e., *L*_*i*_ in (3) and *F*_*i*,*q*_ in (4)), its value can be found by iterating until the value converges [27]. Refer to [9] for the appropriate initial values for the iterations. With this, we define formally the feasibility of a task or a task set as follows.

**Definition 1 (task feasibility).**

Task *τ*_*i*_ with the assignment of *p*_*i*_ and pt_*i*_ is* feasible *↔*R*_*i*_ ≤ *D*_*i*_.

**Definition 2 (task set feasibility).**

Task set Γ with priority ordering PO_*n*_ = 〈*p*_1_^*n*^, *p*_2_^*n*^,…, *p*_|Γ|_^*n*^〉 or task ordering TO_*n*_ = PO_*n*_^−1^ is* feasible *↔ every *τ*_*i*_ in Γ is feasible such that *τ*_*i*_ with *p*_*i*_^*n*^ in PO_*n*_ and pt_*i*_ determined by OPT-ASSIGN-THRESHOLD( ) is feasible.

### 2.2. A Walk-Through Example Task Set

**Figure 1:**
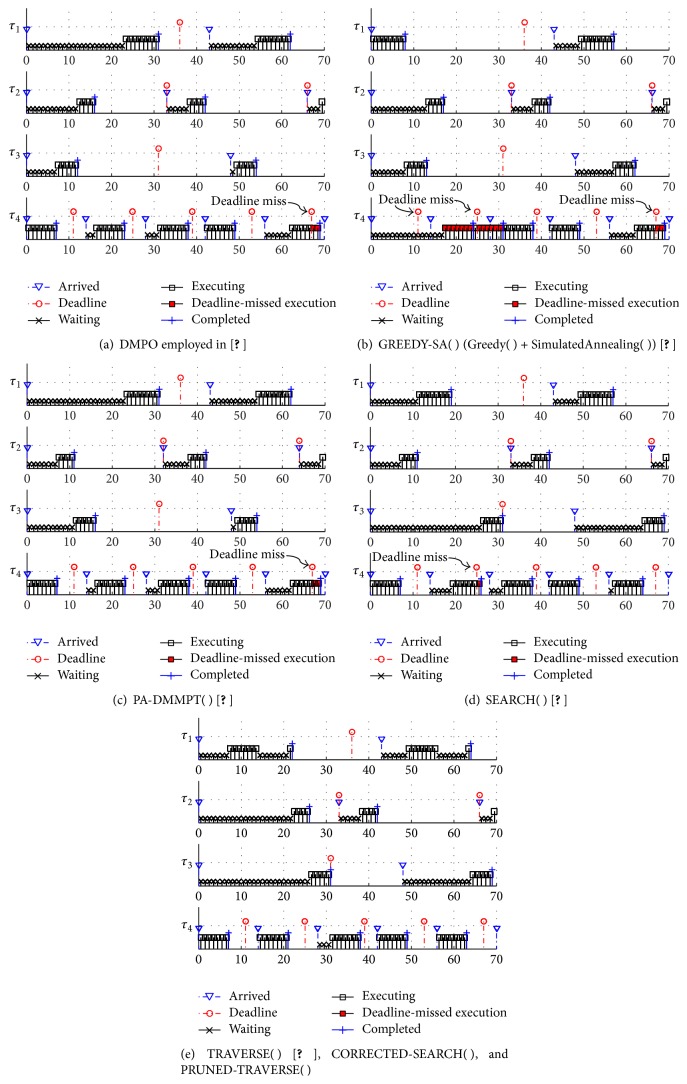
Schedule produced for the walk-through example task set of Table 1 by various priority assignment algorithms. Note that some instances of task *τ*_4_ in (a)~(d) miss their deadlines while every task in (e) does not miss its deadline.

As a walk-through example, we use the task set in Table 1 that is composed of four tasks. The deadline monotonic priority ordering (DMPO) is optimal in the fully preemptive fixed priority scheduling [22] and is so even though there are blockings if there is no jitter [21]. Therefore, the approach of assigning priorities using DMPO and then assigning preemption thresholds using OPT-ASSIGN-THRESHOLD( ) of [1] is widely used in practice, which was also employed in [9] when comparing PTS with other limited preemptive scheduling policies. Since the task indexes of the example task set happen to be in the deadline monotonic decreasing order, the resultant priority ordering of DMPO is PO_*D*_ = 〈1,2, 3,4〉. However, as shown in Table 1, this priority ordering makes task *τ*_4_ miss its deadline since (*R*_4_ = 14)>(*D*_4_ = 11).  Figure 1(a) demonstrates such a deadline miss: the fifth instance of task *τ*_4_ completes at time point 69 while its absolute deadline is (5 − 1) · *T*_4_ + *D*_4_ = 67. Note that the worst-case response time cannot be obtained at the critical instant [29] when there is a nonpreemptiveness of tasks [9].

On the other hand, [3, 24] proposed heuristic priority assignment algorithms for PTS. Reference  [3] proposed an algorithm that combines Greedy( ) and SimulatedAnnealing( ), which we refer to GREEDY-SA( ) in this paper. Reference  [24] proposed PA-DMMPT( ), which means priority assignment algorithm assuming Deadline Monotonic and Maximum Preemption Threshold for the remaining tasks in the unassigned task set. The resultant priority ordering of GREEDY-SA( ) and PA-DMMPT( ) is, respectively, PO_*G*_ = 〈4,2, 3,1〉 and PO_PA_ = 〈1,3, 2,4〉 as shown in Table 1. These priority orderings also make task *τ*_4_ miss its deadline as shown in Table 1.

Figures 1(b) and 1(c) demonstrate such deadline misses of task *τ*_4_. In Figure 1(b), the first, the second, and the fifth instances of task *τ*_4_ complete at time points 24, 31, and 69, respectively, while their absolute deadlines are *D*_4_ = 11, (2 − 1) · *T*_4_ + *D*_4_ = 25, and (5 − 1) · *T*_4_ + *D*_4_ = 67, respectively. In Figure 1(c), the fifth instance of task *τ*_4_ completes at time point 69 while its absolute deadline is (5 − 1) · *T*_4_ + *D*_4_ = 67. As such, widely used DMPO or heuristic priority assignment algorithms may not produce a feasible priority assignment while there is actually one, as will be shown in Section 3.1. This motivated our work.

## 3. Previously Proposed Optimal Priority Assignment Algorithms for PTS

This section analyzes previously proposed optimal priority assignment algorithms for PTS: TRAVERSE( ) [13] and SEARCH( ) [1].

### 3.1. TRAVERSE( ) Algorithm

Algorithm 1(a) shows the pseudo code for TRAVERSE( ). As shown in Algorithm 1(a), TRAVERSE( ) depends on its recursive subroutine, _TRAVERSE( ), which has three parameters: (1)* prio*, the next priority to assign, (2)* UnAssigned*, the set of tasks that are waiting for priority assignment, and (3)  Γ, the set of total tasks (line (3)). _TRAVERSE( ) assigns preemption thresholds by calling OPT-ASSIGN-THRESHOLD( ) of [1] when a complete priority ordering is generated (line (4)). When any preemption threshold assignment is not feasible, OPT-ASSIGN-THRESHOLD( ) returns fail. With this, returning at the last lines (line (9)) happens when a specific priority ordering is not feasible.

The remaining part is composed of a loop that recursively invokes _TRAVERSE( ): each task in* UnAssigned* at line (5) is assigned the priority* prio* at line (6) and remaining unassigned tasks (*UnAssigned* − {*τ*_*i*_}) are recursively assigned *prio* + 1 at line (7). Note that the recursive invocation of _TRAVERSE( ) returns only when the return value of the recursive invocation is* success* (line (7)). We call this priority assignment as the* tentative* priority assignment since if the return value of the recursion is* fail*, the next task in* UnAssigned* is tried for assigning priority* prio*.

**Figure 2:**
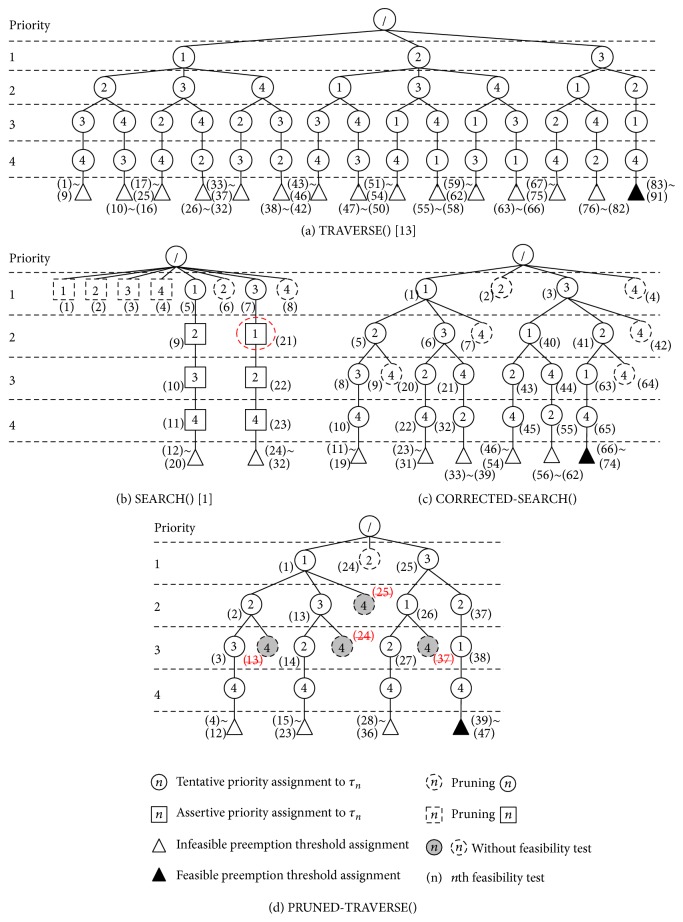
Priority assignment trees for the walk-through example task set of Table 1.

With such tentative priority assignments, _TRAVERSE( ) recursively generates a priority assignment tree for the set of tasks with task orderings from the lowest priority to the highest priority, which is similar to the priority permutation tree of [21].  Figure 2(a) shows the priority assignment tree of _TRAVERSE( ) for the walk-through example task set of Table 1.   In Figure 2, each solid-lined circle node represents a tentative priority assignment to a task and its depth corresponds to its assigned priority. Each triangle node represents an invocation of OPT-ASSIGN-THRESHOLD( ): white one for infeasible (failed) assignment return and black one for feasible (successful) assignment return. Each path from the root to a leaf node corresponds to a possible priority ordering. Each number with round braces “(*n*)” besides a node represents *n*th feasibility test performed for the operation of the node.

In Figure 2(a), 15 complete task orderings TO_1_ = 〈1,2, 3, 4〉, TO_2_ = 〈1,2, 4,3〉,…,  TO_15_ = 〈3,2, 1,4〉 were generated. The last task ordering TO_15_ = TO_*T*_ = 〈3,2, 1,4〉 has a dangled black triangle node, which indicates a feasible preemption threshold assignment. Task ordering TO_*T*_ corresponds to the priority ordering PO_*T*_ = TO_*T*_^−1^ = 〈3,2, 1,4〉 for TRAVERSE( ) of Table 1. As shown in Table 1, TRAVERSE( ) makes no tasks miss their deadlines, which is also demonstrated in Figure 1(e). While [13] did not formally prove the optimality of TRAVERSE( ), we can easily see that TRAVERSE( ) is optimal since it traverses all possible priority orderings in PO^Γ^ until it finds a feasible priority ordering. Besides, it is a simple application of traverse_orderings( ) [21], which is for fully preemptive fixed priority scheduling and its optimality was proved.

However, TRAVERSE( ) is very inefficient since it generates |Γ|! tentative priority orderings in the worst-case, each of which requires maximally |Γ|^2^ feasibility tests [3] via OPT-ASSIGN-THRESHOLD( ). For example, the priority assignment tree of Algorithm 3(a) requires 91 feasibility tests as the numbers besides triangle nodes indicate. Such a large number of feasibility tests can be significantly reduced in our proposed algorithm in Section 5.

### 3.2. SEARCH( ) Algorithm

Algorithm 1(b) shows the pseudo code for SEARCH( ), which becomes exactly the same as TRAVERSE( ) if we remove lines (5)~(13) and replacing* RefinedList* with* UnAssigned* at line (14). The additional part of SEARCH( ) is composed of one loop (lines (5)~(11)) and refining* UnAssigned* to prepare* RefinedList* (lines (12) and (13)). In contrast to the priority assignment loop of TRAVERSE( ), this additional first loop in SEARCH( ) assigns priority* prio* to task *τ*_*i*_ only when *Delay*_*i*_ (calculated at line (6)) is not larger than zero (line (7)) and unconditionally returns from the recursion (line (9)). We call this priority assignment in the first loop as the* assertive* priority assignment since once priority* prio* is assigned to *τ*_*i*_, the other unassigned tasks are not tested for assigning priority* prio*.

The second loop of SEARCH( ) (lines (14)~(18)) performs the tentative priority assignment as TRAVERSE( ) but it works for* RefinedList* instead of* UnAssigned*. The* RefinedList* is generated by sorting tasks in* UnAssigned* in the ascending order of *Delay*_*i*_ (line (12)) and eliminating infeasible tasks even with the highest preemption threshold assignment (line (13)).

With the assertive and tentative priority assignments, _SEARCH( ) generates a priority assignment tree like Figure 2(b), which is for the walk-through example task set of Table 1. In the tree, each solid-lined square node represents an assertive priority assignment to a task while each dashed-lined node represents pruning a priority assignment to a task. In Figure 2(b), two complete task orderings were generated: 〈1,2, 3,4〉 and 〈3,1, 2,4〉. Both orderings failed to assign feasible preemption thresholds to tasks as the dangled white triangle nodes indicate. The last task ordering TO_*S*_ = 〈3,1, 2,4〉 corresponds to the priority ordering PO_*S*_ = TO_*S*_^−1^ = 〈2,3, 1,4〉 for SEARCH( ) of Table 1. As shown in Table 1, SEARCH( ) makes task *τ*_4_ miss its deadline since (*R*_4_ = 14)>(*D*_4_ = 11).  Figure 1(d) demonstrates such a deadline miss of task *τ*_4_ where the second instance of task *τ*_4_ completes at time point 26 while its absolute deadline is *T*_4_ + *D*_4_ = 25. However, the walk-through example task set has a feasible priority ordering PO_*T*_ = 〈3,2, 1,4〉 from TRAVERSE( ) algorithm as we have shown in Section 3.1. This clearly shows SEARCH( ) is not an optimal priority assignment algorithm for PTS.

If we compare the priority assignment trees of TRAVERSE( ) and SEARCH( ) in Figures 2(a) and 2(b), we can see that TO_*S*_ = 〈3,1, 2,4〉 is the 13th task ordering generated by TRAVERSE( ). The reason why SEARCH( ) failed is due to the assertive priority assignment of *p*_1_^*S*^ ← 2 with the 21st feasibility test, which we marked with a red dashed-line circle in Figure 2(b). We correct SEARCH( ) in the next section.

## 4. Corrected SEARCH( ) Algorithm

In this section, we propose CORRECTED-SEARCH( ) algorithm that corrects SEARCH( ) algorithm. We first develop a theorem that is required in correcting SEARCH( ).

**Lemma 3 .**

Under PTS, if priority *p*_*i*_ of task *τ*_*i*_ is fixed, its worst-case response time *R*_*i*_ does not decrease when its preemption threshold pt_*i*_ is lowered.

**Proof**

While *R*_*i*_ is calculated from (1)~(6), pt_*i*_ is only included in (4) for calculating *F*_*i*,*q*_, specifically in the last term of the right side of (4): ∑_∀*j*,*p*_*j*_>pt_*i*__(⌈*F*_*i*,*q*_/*T*_*j*_⌉ − (1 + ⌊*S*_*i*,*q*_/*T*_*j*_⌋)) · *C*_*j*_. We refer to this term as *I*_*i*,*q*_^*F*^ for the convenience of proving. *I*_*i*,*q*_^*F*^ is the summation of the interference time by task *τ*_*j*_ such that ∀*j*, *p*_*j*_ > pt_*i*_ after *S*_*i*,*q*_. Accordingly, the set of tasks that can interfere task *τ*_*i*_ with the lower pt_*i*_ is always the superset of the set of such tasks with the higher pt_*i*_ value. Consequently, *I*_*i*,*q*_^*F*^ does not decrease with lower pt_*i*_. From (1), *R*_*i*_ monotonically increases with respect to to *F*_*i*,*q*_ and *F*_*i*,*q*_ is proportional to *I*_*i*,*q*_^*F*^ from (4). Therefore, *R*_*i*_ is also proportional to *I*_*i*,*q*_^*F*^ and thus *R*_*i*_ does not decrease with lower pt_*i*_.

**Theorem 4 .**

Under PTS, if task *τ*_*i*_ with priority *p*_*i*_^*n*^ in priority ordering PO_*n*_ and the highest preemption threshold pt_*i*_ (pt_*i*_ = |Γ|) is infeasible, task set Γ with PO_*n*_ is also infeasible.

**Proof**

From Definition 1, task *τ*_*i*_ is feasible if and only if *R*_*i*_ ≤ *D*_*i*_. From Lemma 3, if *p*_*i*_ is fixed (as *p*_*i*_^*n*^), pt_*i*_ = |Γ| guarantees the minimal *R*_*i*_ for *τ*_*i*_. Accordingly, if task *τ*_*i*_ with fixed *p*_*i*_ and pt_*i*_ = |Γ| is infeasible, no other preemption threshold assignment can make *R*_*i*_ reduced, that is, *τ*_*i*_ feasible. From Definition 2, Γ with PO_*n*_ is feasible if and only if all tasks with PO_*n*_ in Γ are feasible. This proves the theorem.

Algorithm 2(a) shows the pseudo code of CORRECTED-SEARCH( ), which corrects SEARCH( ) in Algorithm 1(b) as follows:

(i) deletion of the first loop of the assertive priority assignment and sorting tasks (lines (5)~(12) of Algorithm 1(b)),
(ii) replacement of* SortedList* with* UnAssigned* (line (13) of Algorithm 1(b)),
(iii) adding “pt_*i*_ ← *prio*;” after “*p*_*i*_ ← *prio*;” (line (15) of Algorithm 1(b)),
(iv) replacement of OPT-ASSIGN-THRESHOLD(Γ) at line (4) with RESTORING-OPT-ASSIGN-THRESHOLD(Γ) of Algorithm 2(b).

First, CORRECTED-SEARCH( ) does not employ the first loop of SEARCH( ), the assertive priority assignment, which is the main cause of the nonoptimality of SEARCH( ) as shown in the previous section. If we change the assertive priority assignment to a tentative priority assignment by replacing line (9) with line (16) in Algorithm 1(b), it just induces additional feasibility tests (due to invocation of operation WCRT( )) when a given task set is indeed infeasible. In other words, the first loop just becomes a performance bottleneck even though it is corrected and thus we remove it.

Second, without the first loop of SEARCH( ), *Delay*_*i*_ is not calculated and thus the task sorting of line (12) of Algorithm 1(b) is not also employed. Note that the second loop of SEARCH( ) is exactly the same as TRAVERSE( ) except that it works for* RefinedList* instead of* UnAssigned*. From Theorem 4, pruning at line (5) that makes* RefinedList* is valid. With this, we can easily see that CORRECTED-SEARCH( ) is optimal since TRAVERSE( ) is optimal.

Third, we assign task *τ*_*i*_ preemption threshold pt_*i*_ as the same value of its priority at line (8) whenever its priority *p*_*i*_ is assigned (line (7)). This is to make sure that any priority assigned task is preemptable by higher priority tasks. For the feasibility test for refining* UnAssigned* by subroutine Refine( ) at line (5) to work correctly, it should be guaranteed that any task in* UnAssigned* can preempt the preassigned lower priority tasks in Γ − *UnAssigned*. SEARCH( ) does not need this preemption threshold assignment due to WCRT( ) invocation at line (6) of Algorithm 1(b) where every task's preemption threshold is set as its priority.

Finally, RESTORING-OPT-ASSIGN-THRESHOLD( ) in Algorithm 2(b) restores the preemption threshold of each task to its priority in the case of the failed (infeasible) preemption threshold assignment return. Without this correction, after OPT-ASSIGN-THRESHOLD( ) has assigned preemption thresholds to tasks, preemption threshold values of tasks are contaminated and thus subroutine Refine( ) at line (5) cannot work correctly.

Figure 2(c) shows the priority assignment tree of CORRECTED-SEARCH( ) for the walk-through example task set of Table 1. In the figure, six complete task orderings were generated. The last task ordering TO_*C*_ = 〈3,2, 1,4〉 is the same as TO_*T*_, and the resultant feasible task ordering of TRAVERSE( ). CORRECTED-SEARCH( ) requires 74 feasibility tests while TRAVERSE( ) requires 91 feasibility tests as the numbers besides triangle nodes indicate in Figures 2(a) and 2(c). As such, it is obvious that the overall performance of CORRECTED-SEARCH( ) is much better than that of TRAVERSE( ) since CORRECTED-SEARCH( ) prunes infeasible paths while TRAVERSE( ) does not. We develop the more performance enhanced algorithm in the next section.

## 5. PRUNED-TRAVERSE( ) Algorithm

SEARCH( ) is not optimal since it prunes even feasible priority orderings. As such, pruning exhaustively without harming the optimality is important. In this section, we propose our optimal priority assignment algorithm for PTS, which we named PRUNED-TRAVERSE( ). We first develop required theorems for our proposed algorithm.

**Lemma 5 .**

Under any priority assignment algorithm that assigns distinctive priorities to tasks for PTS, if the priority *p*_*i*_ of a task *τ*_*i*_ is lowered to *p*_*i*_′ (*p*_*i*_′ < *p*_*i*_), there exists at least one lower priority task *τ*_*j*_ (*p*_*j*_ < *p*_*i*_) that heightens its priority *p*_*j*_ to *p*_*j*_′ with *p*_*j*_′ > *p*_*i*_′.

**Proof**

The lemma can be easily proved by observing task orderings in priority assignment trees like Figure 2(a). If task *τ*_*i*_ is moved to a lower priority level (the higher place), at least one of the lower priority tasks should be moved to a higher priority level (the lower place) since the total priority levels are fixed.

**Theorem 6 .**

Under PTS, if preemption threshold pt_*i*_ of task *τ*_*i*_ is fixed, its worst-case response time *R*_*i*_ does not decrease when its priority *p*_*i*_ is lowered.

**Proof**

For the convenience of proving, we refer to the last terms of the right sides of (4) and (5) as *I*_*i*,*q*_^*F*^ and *I*_*i*,*q*_^*S*^, respectively. We prove the theorem by contradiction. Suppose that *R*_*i*_ of task *τ*_*i*_ decreases to *R*_*i*_′ when its priority *p*_*i*_ is lowered to *p*_*i*_′: *R*_*i*_′ < *R*_*i*_ and *p*_*i*_′ < *p*_*i*_. For *R*_*i*_ to decrease, *F*_*i*,*q*_ should decrease from (1). For *F*_*i*,*q*_ to decrease, *S*_*i*,*q*_ or *I*_*i*,*q*_^*F*^ should decrease from (4). Since the condition for calculating *I*_*i*,*q*_^*F*^ is *p*_*j*_ > pt_*i*_, the change of priority *p*_*i*_ does not affect *I*_*i*,*q*_^*F*^. Therefore, *S*_*i*,*q*_ should decrease to *S*_*i*,*q*_′ such that *S*_*i*,*q*_ − *S*_*i*,*q*_′ > 0. Then, from (5), (*B*_*i*_ + *I*_*i*,*q*_^*S*^)−(*B*_*i*_′ + *I*_*i*,*q*_^*S*^′) > 0 follows where *B*_*i*_′ and *I*_*i*,*q*_^*S*^′ are changed values of *B*_*i*_ and *I*_*i*,*q*_^*S*^ due to the lower priority. Then, (*B*_*i*_ − *B*_*i*_′)>(*I*_*i*,*q*_^*S*^′ − *I*_*i*,*q*_^*S*^) follows.

From Lemma 5, if *p*_*i*_ is lowered to *p*_*i*_′ there exists at least one lower priority task *τ*_*j*_ (*p*_*j*_ < *p*_*i*_) that heightens its priority *p*_*j*_ to *p*_*j*_′ such that *p*_*j*_′ > *p*_*i*_′. Let the set of such additionally introduced higher priority tasks after the priority of task *τ*_*i*_ is lowered be *AddedHP*_*i*_ = {*τ*_*j*_∣*p*_*j*_ < *p*_*i*_  and  *p*_*j*_′ > *p*_*i*_′}. The maximum possible value of (*B*_*i*_ − *B*_*i*_′) is achieved when *B*_*i*_′ = 0, which infers that at least one task in *AddedHP*_*i*_ contributes in making *B*_*i*_. Let such a task in *AddedHP*_*i*_ be task *τ*_*k*_; that is *B*_*i*_ = *C*_*k*_. Then, max(*B*_*i*_ − *B*_*i*_′) = *C*_*k*_ follows. On the other hand, from (5), it follows that *I*_*i*,*q*_^*S*^′ − *I*_*i*,*q*_^*S*^ = ∑_∀*j*,*p*_*j*_∈*AddedHP*_*i*__(1 + ⌊*S*_*i*,*q*_′/*T*_*j*_⌋) · *C*_*j*_ ≥ *C*_*k*_. Then, (*B*_*i*_ − *B*_*i*_′)≤(*I*_*i*,*q*_^*S*^′ − *I*_*i*,*q*_^*S*^) follows. This is a contradiction, which proves the theorem.

**Theorem 7 .**

Under PTS, if task *τ*_*i*_ with priority *p*_*i*_^*n*^ in priority ordering PO_*n*_ and the highest preemption threshold pt_*i*_ = |Γ| is infeasible, task set Γ with another priority ordering PO_*m*_ that assigns task *τ*_*i*_ the lower priority *p*_*i*_^*m*^ (such that *p*_*i*_^*m*^ < *p*_*i*_^*n*^) is also infeasible.

**Proof**

Let the worst-case response time of task *τ*_*i*_ with pt_*i*_ = |Γ| and *p*_*i*_^*n*^ be *R*_*i*_. Let the worst-case response time of task *τ*_*i*_ with pt_*i*_ = |Γ| and *p*_*i*_^*m*^ be *R*_*i*_′. From Lemma 3, both *R*_*i*_ and *R*_*i*_′ are the minimal possible worst-case response time with each given priority. From Theorem 6, *R*_*i*_′ ≥ *R*_*i*_ follows. Since task *τ*_*i*_ with *p*_*i*_^*n*^ and pt_*i*_ = |Γ| is infeasible, *R*_*i*_ > *D*_*i*_ follows from Definition 1. Therefore, *R*_*i*_′ > *D*_*i*_ follows. Accordingly, task *τ*_*i*_ with *p*_*i*_^*m*^ and pt_*i*_ = |Γ| is infeasible. Consequently, from Theorem 4, task set Γ with PO_*m*_ is also infeasible.

Now we propose PRUNED-TRAVERSE( ) that extends TRAVERSE( ) by exhaustively pruning infeasible paths. PRUNED-TRAVERSE( ) prunes such priority ordering PO_*n*_ that assigns task *τ*_*i*_ priority *p*_*i*_^*n*^ when the following condition is true:

(7) $$\begin{array}{c} \text{C}1\vee \text{C}2, \end{array}$$

where C1 is pt_*i*_ = |Γ|∧*R*_*i*_ > *D*_*i*_, and C2 is *p*_*i*_^*n*^ < max⁡{*p*_*i*_∣C1}.

Apparently, Condition C1 is from Theorem 4 and Condition C2 is from Theorem 7.

Algorithm 3 shows the pseudo code for PRUNED-TRAVERSE( ). Like TRAVERSE( ), PRUNED-TRAVERSE( ) depends on its subroutine, _PRUNED-TRAVERSE( ), which has the same parameters as _TRAVERSE( ):* prio*,* UnAssigned*, and Γ (line (9)). Like _TRAVERSE( ), _PRUNED-TRAVERSE( ) assigns priorities to tasks from the lowest priority 1 to the highest priority |Γ|: it assigns each task *τ*_*i*_ in* UnAssigned* (line (10)) priority* prio* (line (12)) and the remaining unassigned tasks (*UnAssigned* − {*τ*_*i*_}) are recursively assigned priority *prio* + 1 at line (21).

Unlike TRAVERSE( ), PRUNED-TRAVERSE( ) first sorts tasks in a deadline monotonic decreasing order since DMPO also works well in many cases (line (2)). PRUNED-TRAVERSE( ) introduces for task *τ*_*i*_ new attribute *infeasiblePrioMax*_*i*_ = max⁡{*p*_*i*_∣C1}, which is the right side of the Condition C2. It initializes *infeasiblePrioMax*_*i*_ as zero at line (4).

Unlike _TRAVERSE( ), _PRUNED-TRAVERSE( ) needs to perform a feasibility test for task *τ*_*i*_ (getting *R*_*i*_) while assigning each priority in order to prune infeasible paths. For this, we need to make sure that all tasks in* UnAssigned* have higher priorities than the priorities of the previously priority assigned tasks. For this, PRUNED-TRAVERSE( ) assigns the highest priority |Γ| to all unassigned tasks initially at line (5). _PRUNED-TRAVERSE( ) also does so conditionally at line (26) whenever the next priority (*prio* + 1) assignment fails at line (25). Preemption thresholds of all tasks are also initialized as the same value of their priorities at line (6).

Before assigning task *τ*_*i*_ priority* prio* (line (12)), _PRUNED-TRAVERSE( ) first prunes any infeasible priority ordering at line (11) if* prio* is smaller than *infeasiblePrioMax*_*i*_, which is Condition C2. Condition C1 is applied at lines (13) and (19) and the priority ordering with Condition C1 is pruned at line (22). Line (20) is for restoring the priority of the pruned task. Line (21) is for updating *infeasiblePrioMax*_*i*_. If task *τ*_*i*_ is assigned priority* prio* without being pruned, its preemption threshold is assigned the same as its priority at line (24), without which PTS becomes the fully nonpreemptive scheduling (NPS) due to the highest preemption threshold assignment at line (13).

Once* prio* of the maximum priority |Γ| is assigned (line (14)), which means that a complete priority ordering is generated, _PRUNED-TRAVERSE( ) assigns preemption thresholds by invoking RESTORING-OPT-ASSIGN-THRESHOLD( ) in Algorithm 2(b). Note that there are two differences with TRAVERSE( ) in assigning preemption thresholds: (1) invoking RESTORING-OPT-ASSIGN-THRESHOLD( ) instead of OPT-ASSIGN-THRESHOLD( ) and (2) assigning preemption thresholds to tasks once the maximum priority is assigned instead once* UnAssinged* is { }. (1) is for the proper pruning operation as we explained in proposing CORRECTED-SEARCH( ) in Section 4. (2) is for reducing one recursive function call for a performance benefit: one recursive function call is reduced for each complete priority ordering in PRUNED-TRAVERSE( ) compared to TRAVERSE( ) or CORRECTED-SEARCH( ).

When any preemption threshold assignment is not feasible, RESTORING-OPT-ASSIGN-THRESHOLD( ) returns fail. In that case, *infeasiblePrioMax*_*i*_ is set to |Γ| at line (16). This is because the fail return of RESTORING-OPT-ASSIGN-THRESHOLD( ) infers that the highest priority assigned task *τ*_*i*_ is not feasible due to some blocking task whose preemption threshold should be raised for it to be feasible. The recursive invocation of _PRUNED-TRAVERSE( ) returns only when the return value of the recursive invocation is* success* (line (25)). With this, returning at the last lines (line (28)) happens when a specific ordering is not feasible.

_PRUNED-TRAVERSE( ) requires careful restorations of priorities and preemption thresholds of tasks, which are repetitively and tentatively assigned. Since SEARCH( ) and CORRECTED-SEARCH( ) prune infeasible paths within subroutine Refine( ), such restoration is easier and less error-prone. However, PRUNED-TRAVERSE( ) prunes infeasible paths as earlier as possible for the better efficiency and thus requires careful restoring operations. Priorities are restored before pruning at line (20) and after the failed priority assignment at line (26). Preemption thresholds are restored in RESTORING-OPT-ASSIGN-THRESHOLD( ) at line (15). Assigning the preemption threshold as same as the priority of the priority assigned task at line (24) is also important for the proper pruning.

Now we prove the optimality of PRUNED-TRAVERSE( ).

**Theorem 8 .**

PRUNED-TRAVERSE( ) is optimal for PTS: it finds a feasible scheduling attributes assignment if there exists one.

**Proof**

PRUNED-TRAVERSE( ) extends TRAVERSE( ) by pruning infeasible priority orderings with Condition C1 or C2. Condition C1 is valid from Theorem 4 and condition C2 is valid from Theorem 7. Consequently, since TRAVERSE( ) is optimal, PRUNED-TRAVERSE( ) is optimal.

Figure 2(d) shows the priority assignment tree of PRUNED-TRAVERSE( ) for the walk-through example task set of Table 1. In the figure, a gray dashed-lined circle represents pruning a priority ordering without any feasibility test, which is achieved by application of Condition C2 from Theorem 7. By comparing Figures 2(c) and 2(d), we can easily see this additional pruning helps much in reducing the number of feasibility tests: the feasibility tests of (7), (21)~(39), (55)~(62) in Figure 2(c) do not happen in Figure 2(d). We can also see that the earlier pruning with condition C1 of PRUNED-TRAVERSE( ) instead of using subroutine Refine( ) of CORRECTED-SEARCH( ) reduces the number of feasibility tests: the feasibility tests of (4), (42), (64) in Figure 2(c) do not happen in Figure 2(d).

The resultant last task ordering TO_*P*_ = 〈3,2, 1,4〉 is the same as TO_*C*_ and TO_*T*_, which are the resultant feasible task ordering of CORRECTED-SEARCH( ) and TRAVERSE( ), respectively. PRUNED-TRAVERSE( ) produces four complete priority orderings and requires 47 feasibility tests while CORRECTED-SEARCH( ) produces six complete priority orderings and requires 74 feasibility tests. As such, it is obvious that the overall performance of PRUNED-TRAVERSE( ) is much better than that of CORRECTED-SEARCH( ) since PRUNED-TRAVERSE( ) prunes more infeasible paths than CORRECTED-SEARCH( ) exploiting Theorem 7 and the earlier pruning. We show the empirical performance comparison results in Section 7.

## 6. Complexity

Let *n* = |Γ| and *E* the nonpolynomial [20] complexity of the feasibility test (calculating *R*_*i*_ from (1)~(6)). Since the pruning operation is conditional, all these algorithms in the worst-case produce *n*! priority orderings of PO^Γ^, each of which requires *O*(*n* ^2^) feasibility tests [3] via OPT-ASSIGN-THRESHOLD( ). With this, TRAVERSE( ) has the complexity of *O*(*E* · *n*!·*n* ^2^).

On the other hand, each pruning operation of CORRECTED-SEARCH( ) and PRUNED-TRAVERSE( ) requires one feasibility test for each tentative priority assignment or pruning (circle) node. The number of circle nodes in a priority assignment tree in the worst-case is *n* + *n* · (*n* − 1) + *n* · (*n* − 1)·(*n* − 2)+⋯+*n*!, which is *O*(*n*!). Therefore, the worst-case complexity of CORRECTED-SEARCH( ) and PRUNED-TRAVERSE( ) is *O*(*E* · *n*!·*n* ^2^) + *O*(*E* · *n*!) = *O*(*E* · *n*!·*n* ^2^), which is the same as that of TRAVERSE( ). However, the pruning operation obviously works as a branch and bound mechanism and derives much better performances in most cases as the following empirical comparison shows.

## 7. Empirical Performance Evaluations

We set the performance metrics of priority assignment algorithms for PTS as (1) schedulability as the ratio of feasible task sets and (2) actual runtimes for executing algorithms. The experiments for getting the actual runtimes of the algorithms were done on Intel Core i7-4770, 3.40 GHz with 8 GB RAM. We set the total utilization of each task set as *U* = 0.9. This utilization represents high-demanding workloads, which makes the feasibility of a given task set be greatly dependent on a proper priority assignment algorithm. Note that the utilization bound *U*_*B*_ under the rate monotonic scheduling (RMS) [29] when |Γ| = *n* is $U_{B}=n\cdot \left(\sqrt[{n}]{2}-1\right).$ For example, if |Γ| = 10 and *U* ≤ 0.72 ($≅10\cdot (\sqrt[{10}]{2}-1$) = *U*_*B*_), we even do not need PTS if task deadlines are the same as periods since the fully preemptive fixed priority scheduling with DMPO always makes the task set feasible.

We generated each task set in the same manner as [9]. Specifically, for a given total utilization *U* = 0.9, we generated each task's utilization *u*_*i*_ using UUniFast [30] algorithm. For each task *τ*_*i*_, we generated *C*_*i*_ as a random integer uniformly distributed in the interval [100,500], *T*_*i*_ = *C*_*i*_/*u*_*i*_, and *D*_*i*_ as a random integer uniformly distributed in the interval [*C*_*i*_ + 0.5 · (*T*_*i*_ − *C*_*i*_), *T*_*i*_]. For each experiment with a specific parameter setting, we generated 2,000 task sets. To focus on the effectiveness of pruning operations of PRUNED-TRAVERSE( ), each task set was ordered in the deadline monotonic decreasing order. Since TRAVERSE( ) took too much time when the number of tasks is large, we applied timeout for executing TRAVERSE( ) when |Γ| ≥ 10. The actual runtimes of TRAVERSE( ) with timeout were set as the real actual runtime of TRAVERSE( ) when a task set was not feasible.

### 7.1. Schedulability

**Figure 3:**
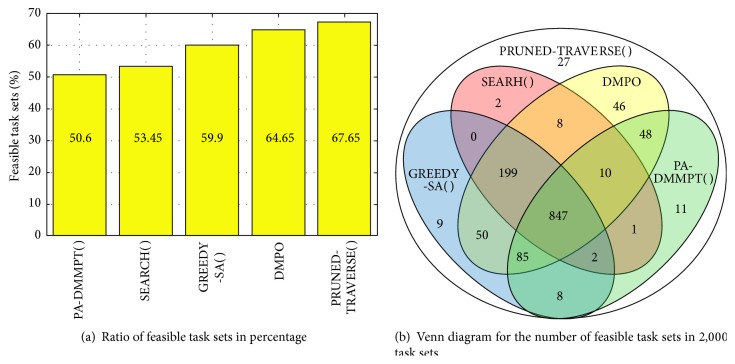
Schedulability with *U* = 0.9 and |Γ| = 10.

Figure 3 shows the schedulability results with *U* = 0.9 and |Γ| = 10.  Figure 3(a) shows the percentage ratio of feasible task sets and Figure 3(b) shows the Venn diagram for the number of feasible task sets. The ratio of feasible task sets was calculated as the number of feasible task sets divided by the total number of generated task sets. The resultant feasible task set ratios of TRAVERSE( ), CORRECTED-SEARCH( ), and PRUNED-TRAVERSE( ) were exactly the same and thus we omitted the results of TRAVERSE( ) and CORRECTED-SEARCH( ) in Figure 3.

Figure 3(a) clearly shows that the schedulability performance results of PA-DMMPT( ) [24], SEARCH( ) [1], GREEDY-SA( ) [3], DMPO [9], and PRUNED-TRAVERSE( ) are uniformly improved in order. Specifically, PA-DMMPT( ), SEARCH( ), GREEDY-SA( ), DMPO, and PRUNED-TRAVERSE( ) could, respectively, schedule 50.6%, 53.45%, 59.9%, 64.65%, and 67.65%. Note that PRUNED-TRAVERSE( ) outperforms than any other priority assignment algorithms, which clearly shows that the other algorithms are nonoptimal.

Figure 3(b) shows the Venn diagram for the number of feasible task sets in 2,000 task sets. As shown, PRUNED-TRAVERSE( ) could schedule 27 task sets that could not be scheduled by any other existing heuristic priority assignment algorithms. It is notable that each heuristic algorithm could schedule some task sets that could not be scheduled by the other heuristic algorithms. For example, SEARCH( ) could schedule two task sets that could not be scheduled by the other heuristic algorithms.

DMPO is a very efficient algorithm that requires almost no implementation efforts as well as computation time burden. Accordingly, it is practical to combine DMPO with another heuristic algorithm. Figure 3(b) shows that SEARCH( ), GREEDY-SA( ), and PA-DMMPT( ), respectively, could schedule 5 (2 + 0 + 2 + 1), 19 (9 + 8 + 2 + 0), and 22 (11 + 1 + 2 + 8) task sets that could not be schedule by DMPO. On the other hand, DMPO + SEARCH( ), DMPO + GREEDY-SA( ), and DMPO + PA-DMMPT( ), respectively, could schedule 64.9%, 65.6%, and 65.75%. With this, we conclude that PA-DMMPT( ) is the most effective heuristic priority assignment algorithm as the best candidate to be combined with DMPO. Therefore, we compare its actual runtimes with our proposed algorithms in the next subsection. Note that any DMPO combined heuristic algorithm cannot become optimal due to the existence of task sets that can be scheduled only by PRUNED-TRAVERSE( ) as shown in Figure 3(b).

### 7.2. Actual Runtimes

**Figure 4:**
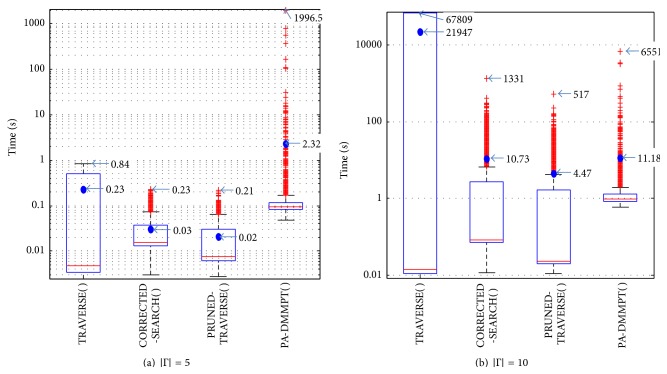
Actual runtimes in seconds when *U* = 0.9.

We compare the actual runtimes for executing optimal priority assignment algorithms and PA-DMMPT( ), which is the most effective heuristic algorithm to be combined with DMPO as shown in the previous section. Figures 4(a) and 4(b) show the actual runtime results in seconds as box plots when *U* = 0.9 for |Γ| = 5 and |Γ| = 10, respectively. *Y* axis is in a log scale to better show the distributions of result values. Each box in a box plot shows data results between 25% and 75% of performance distribution. The middle line within each box shows the median value of the results while the filled circle mark shows the average value of the results.

Figures 4(a) and 4(b) clearly show that the actual runtime performance distribution results of TRAVERSE( ), CORRECTED-SEARCH( ), and PRUNED-TRAVERSE( ) are uniformly improved in order. In Figure 4(a) with |Γ| = 5, the maximum and average runtimes were decreased by 72.6% and 87.0%, respectively, in CORRECTED-SEARCH( ) compared to the TRAVERSE( ) (from 0.84 sec to 0.23 sec and from 0.23 sec to 0.03 sec). PRUNED-TRAVERSE( ) further decreased the maximum and average actual runtime values of CORRECTED-SEARCH( ) by 8.7% and 33.3%, respectively (to 0.21 sec and to 0.02 sec).

Such performance differences become drastically large as the number of tasks |Γ| increases. In Figure 4(b) with |Γ| = 10, the maximum and average actual runtimes of CORRECTED-SEARCH( ) compared to TRAVERSE( ) were decreased by 98.0% and 99.95%, respectively (from 67809 sec to 1331 sec and from 21947 sec to 10.73 sec). Moreover, PRUNED-TRAVERSE( ) decreased the maximum and average values of CORRECTED-SEARCH( ) by 61.2% and 58.3%, respectively (to 517 sec and to 4.47 sec).

On the other hand, we can see the median values of actual runtimes of TRAVERSE( ) were always smaller than any other algorithms. This is because TRAVERSE( ) does not perform any feasibility test for priority assignments that are required by the other algorithms. When a task set is feasible with DMPO, the actual runtimes of TRAVERSE( ) are the same as DMPO.

Figures 4(a) and 4(b) also show that the actual runtimes of PA-DMMPT( ) are much larger than PRUNED-TRAVERSE( ) and even larger than CORRECTED-SEARCH( ). Specifically in Figure 4(b) with |Γ| = 10, the maximum and average actual runtimes of PA-DMMPT( ) compared to PRUNED-TRAVERSE( ) were increased by 1167% and 150% (from 517 sec to 6551 sec and from 4.47 sec to 11.18 sec). This is because PA-DMMPT( ) calculates task blocking limits by generating candidate pairs of start and finish times of tasks. As well investigated in [31], the execution requirements of such an approach that generates scheduling points grow exponentially with an increasing range of task periods. Our experimental task sets were generated in the same manner as [9], where the range of task periods increases as the number of tasks increases.

However, PRUNED-TRAVERSE( ) still cannot be used as an online feasibility test algorithm since its performance is also degraded much as the number of tasks increases. For example, for a task set Γ with *U* = 0.9 and |Γ| = 20, PRUNED-TRAVERSE( ) took 7 hours to determine that the task set was after all infeasible. Nevertheless, the above experimental results clearly show that PRUNED-TRAVERSE( ) outperforms than any other optimal priority assignment algorithms as well as the best effective heuristic priority assignment algorithm for PTS.

## 8. Conclusion

Preemption threshold scheduling (PTS) has been widely accepted in the industrial domain for its effectiveness of scalable real-time embedded system design with the increased real-time schedulability (feasibility). However, without an available optimal scheduling attributes assignment algorithm (optimal in the sense that it is guaranteed to find a feasible scheduling attributes assignment if one exists), we cannot achieve the full benefits of PTS.

Since there exists an optimal and efficient *O*(*n* ^2^) preemption threshold assignment algorithm [1] that operates with fully assigned priority orderings, we need an optimal priority assignment algorithm for PTS. In this paper, we analyzed previously proposed optimal priority assignment algorithms for PTS: TRAVERSE( ) [13] and SEARCH( ) [1]. Using priority assignment trees, we showed the inefficiency of TRAVERSE( ) due to its lack of any pruning operation. We also showed the nonoptimality of SEARCH( ) due to its pruning of even feasible priority orderings.

We developed some theorems for safely and exhaustively pruning infeasible priority ordering paths while assigning priorities to tasks before assigning feasible preemption thresholds for PTS. Using these theorems, we corrected SEARCH( ) and presented CORRECTED-SEARCH( ) algorithm. We also proposed PRUNED-TRAVERSE( ) that enhances the performance of CORRECTED-SEARCH( ) while proving its optimality. Our empirical evaluation results clearly showed the effectiveness of PRUNED-TRAVERSE( ) both in schedulability and actual runtimes compared to any other existing priority assignment algorithms for PTS.

## Figures and Tables

**Algorithm 1:**
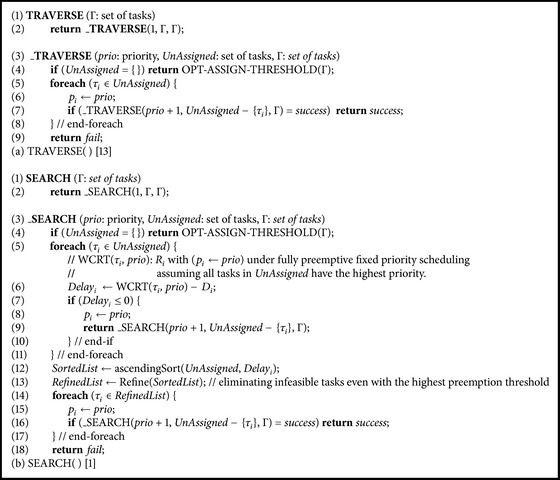
Pseudo code for (a) TRAVERSE( ) [13] and (b) SEARCH( ) [1].

**Algorithm 2:**
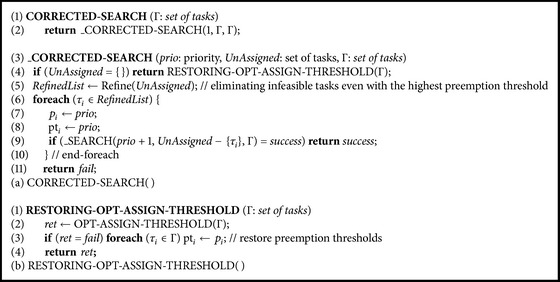
Pseudo code for (a) CORRECTED-SEARCH( ) and (b) RESTORING-OPT-ASSIGN-THRESHOLD( ).

**Algorithm 3:**
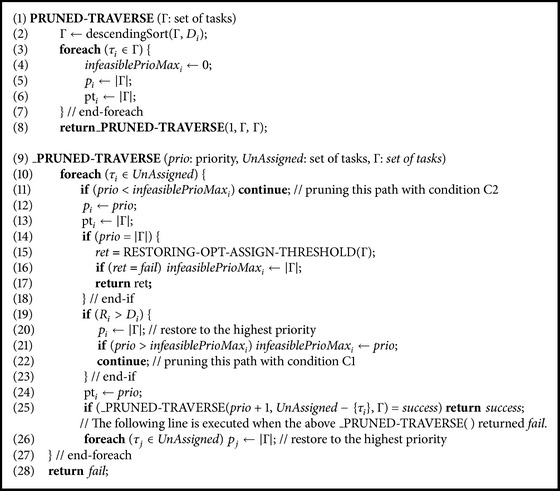
Pseudo code for the proposed PRUNED-TRAVERSE( ) algorithm.

**Table 1:** A walk-through example task set. Bold and italic *R*_*i*_'s represent infeasible response times.

Tasks	*C*_*i*_	*T*_*i*_	*D*_*i*_	DMPO [9]			GREEDY-SA( ) [3]			PA-DMMPT( ) [24]			SEARCH( ) [1]			TRAVERSE( ) [13]		
				*p*_*i*_	pt_*i*_	*R*_*i*_	*p*_*i*_	pt_*i*_	*R*_*i*_	*p*_*i*_	pt_*i*_	*R*_*i*_	*p*_*i*_	pt_*i*_	*R*_*i*_	*p*_*i*_	pt_*i*_	*R*_*i*_
*τ*_1_	8	43	36	1	4	31	4	4	14	1	4	31	2	4	30	3	3	26
*τ*_2_	4	33	33	2	4	30	2	2	23	3	3	25	3	3	25	2	4	30
*τ*_3_	5	48	31	3	3	26	3	3	19	2	4	30	1	4	31	1	4	31
*τ*_4_	7	14	11	4	4	***14***	1	5	***24***	4	4	***14***	4	4	***14***	4	4	11

(i) DMPO: deadline monotonic priority ordering + OPT-ASSIGN-THRESHOLD( ) employed in [9].

(ii) GREEDY-SA( ): Greedy( ) + SimulatedAnnealing( ) proposed in [3].

## Notations

*τ*_*i*_: A task
Γ: The set of tasks {*τ*_1_, *τ*_2_,…, *τ*_|Γ|_}
|Γ|: The number of tasks in task set Γ, the highest priority value
*C*_*i*_: The worst-case execution time of task *τ*_*i*_
*T*_*i*_: The period of task *τ*_*i*_
*D*_*i*_: The relative deadline of task *τ*_*i*_
*p*_*i*_: The priority of task *τ*_*i*_
pt_*i*_: The preemption threshold of task *τ*_*i*_
PO^Γ^: The set of all distinct priority orderings {PO_1_, PO_2_,…, PO_|Γ|!_} in task set Γ
PO_*n*_: A priority ordering, a sequence of task priorities 〈*p*_1_^*n*^, *p*_2_^*n*^,…, *p*_|Γ|_^*n*^〉
*p*_*i*_^*n*^: The priority of task *τ*_*i*_ in priority ordering PO_*n*_
PO_*A*_: The resultant priority ordering generated by priority assignment algorithm ALGORITHM( )
TO_*n*_: A task ordering from the lowest priority to the highest priority, a sequence of task indexes 〈*i*, *j*,…, *k*〉
PO_*n*_^−1^: The inverse mapping of priority ordering PO_*n*_, the task ordering TO_*n*_
TO_*n*_^−1^: The inverse mapping of task ordering TO_*n*_, the priority ordering PO_*n*_
*R*_*i*_: The worst-case response time of task *τ*_*i*_
*L*_*i*_: The longest level-*p*_*i*_ busy period
*B*_*i*_: The worst-case blocking time of task *τ*_*i*_
*S*_*i*,*q*_: The start time of the *q*th instance of task *τ*_*i*_ in *L*_*i*_
*F*_*i*,*q*_: The finish time of the *q*th instance of task *τ*_*i*_ in *L*_*i*_.
